# Higher serum chromium level may be associated with the presentation of depression in patients with metabolic dysfunction-associated fatty liver disease: evidence from NHANES survey

**DOI:** 10.3389/fpsyt.2024.1330283

**Published:** 2024-03-18

**Authors:** Xiuhua Li, Xuezhong Xia, Bolin Jiang, Yao Yao, Fengjiao Ding, Shanyu Qin

**Affiliations:** ^1^ Department of Gastroenterology, The First Affiliated Hospital of Guangxi Medical University, Nanning, China; ^2^ Department of Gastroenterology, Yiyang Central Hospital, Yiyang, China; ^3^ Department of Nursing, Yiyang Central Hospital, Yiyang, China; ^4^ Department of Mental Health, No. 1 Middle School, Yiyang, China

**Keywords:** MAFLD, chromium, depression, NHANES, metabolic disorder

## Abstract

**Background:**

Depressive symptoms are frequently observed in patients with Metabolic Dysfunction-Associated Fatty Liver Disease (MAFLD), a prevalent metabolic disorder that affects many individuals. It is not yet clear whether there is an association between serum chromium levels and depression.

**Objective:**

The purpose of this research was to explore the association between serum chromium level and the manifestation of depression among patients with MAFLD.

**Methods:**

The selection of 1837 patients diagnosed with MAFLD was based on data obtained from the 2017-2018 National Health and Nutrition Examination Survey (NHANES) database in this research. The Patient Health Questionnaire-9 (PHQ-9) was employed to evaluate the severity of depression. The researchers utilized logistic regression models that were weighted for multiple variables to investigate the association between depression and serum chromium levels.

**Results:**

In our study, we found that 8.98% of US adults with MAFLD were suffering from depression at the time of evaluation. In the logistic regression model, serum chromium levels showed an inverse association with depression (OR=0.82, 95%CI: 0.69-0.96; p=0.016), this relationship remained after adjusting for fully confounding factors (OR=0.83, 95%CI: 0.71-0.97; p=0.021), subgroup analyses showed that the association between serum chromium levels and depression existed in relatively high-prevalence of depression groups.

**Conclusion:**

Patients diagnosed with MAFLD have a greater likelihood of experiencing depression, whereas individuals with higher levels of serum chromium are less likely to suffer from depression, and this association persists even after adjusting for other factors. These findings indicate supplementing chromium may be a viable treatment for their depressive symptoms.

## 1 Introduction

With the rapid development of society, metabolic associated fatty liver disease (MAFLD) [formerly known as non-alcoholic fatty liver disease (NAFLD)] has risen in prevalence to a shocking level and placed an enormous burden on health and economy to society, affecting about 1 billion people worldwide (1). MAFLD, put forward by The International Fatty Liver Panel as a fresh nomenclature (2) in April 2020, presents a more comprehensive and precise understanding of the liver through various tests, such as biopsies, imaging scans, and blood markers, as well as accompanying health issues like obesity, diabetes, and metabolic disorders. MAFLD is commonly associated with a plethora of extra-hepatic conditions, such as diabetes, cardiovascular disease, and depression (3, 4), which makes it a high-risk factor for health. There is currently no approved pharmacologic treatment for MAFLD; hence, it is important to better understand the modifiable risk factors for it.

Depression is one of the most widespread chronic medical illnesses, estimated to impact around 5% of the adult population and is among the top causes of disability on a global scale (5). It is characterized by depressed mood, an inability to enjoy life, cognitive impairment, sadness, and physical symptoms, and its etiology remains poorly understood (6). So far, there is no satisfactory therapy for patients with depression (7). Its early onset and frequent recurrences make it a major cause of disability and lead to a heavy economic and health burden for patients (8). Additionally, a recent study identified depression as a risk factor for several physical diseases, such as cancer, cardiovascular diseases, and metabolic disorders (9). Another research investigated associated factors for MAFLD in adults aged 45-79 years and found that the presence of depression was an independent predictor of MAFLD risk (10).

The identification of an association between MAFLD and depression in our study may have implications for how healthcare professionals approach the diagnosis and treatment of individuals affected by both conditions. The association between MAFLD and depression is intricate, and insulin resistance (IR) is believed to have a crucial involvement in the development of both disorders (11, 12). IR is a disordered biological response to insulin stimulation. In addition to the influence of abnormalities on lipid metabolism (13), IR indirectly contributes to MAFLD through inflammation (14). It is also recognized as having a close association with depression (15). Studies suggest a positive association between insulin resistance and depression (16, 17). IR is a predictor of non-response to some antidepressants, it seems to be a state-marker of clinical or subclinical depression, moreover, some insulin-sensitizing interventions are beneficial for the treatment of major depressive disorder (18). Furthermore, a systematic review investigated the antidepressant effects of insulin-sensitizing medications and found that the use of insulin-sensitizing medications had a positive impact on depressive symptoms (19).

Chromium is an essential trace element that plays a significant role in metabolic disorders (20). Individuals with diabetes and obesity have been found to have decreased levels of chromium in their serum compared to those who are healthy (21–23). Some scholars suggest that chromium benefits our health by promoting glycolysis in muscle and fat cells and increasing insulin efficiency in carbohydrate, lipid, and protein metabolism (24–26). Studies on chromium have confirmed its well-described effects on increasing insulin sensitivity in the brain (27) and its anti-inflammatory properties (28). An animal model study showed that chromium played a role in improving insulin signaling, as revealed by its impact on gene expression related to insulin signaling (29). According to one study, chromium picolinate shows promising antidepressant effects in atypical depression (30). However, research examining serum chromium and depression using a subsample from the 2015-2016 US National Health and Nutrition Examination Survey found no significant association between them (13). These contradictory results make it worthwhile to examine the possible association between serum chromium and depression in MAFLD patients. The findings of this investigation can provide evidence regarding whether chromium supplementation is an applicable method for alleviating depression symptoms in MAFLD patients. The main objective of this research is to investigate the association between chromium and depression among a diverse group of American adults diagnosed with MAFLD, ensuring that the sample is representative of the entire nation. The information was gathered from the NHANES survey conducted between 2017 and 2018 to form the dataset.

## 2 Materials and methods

### 2.1 Participants

**Figure 1:**
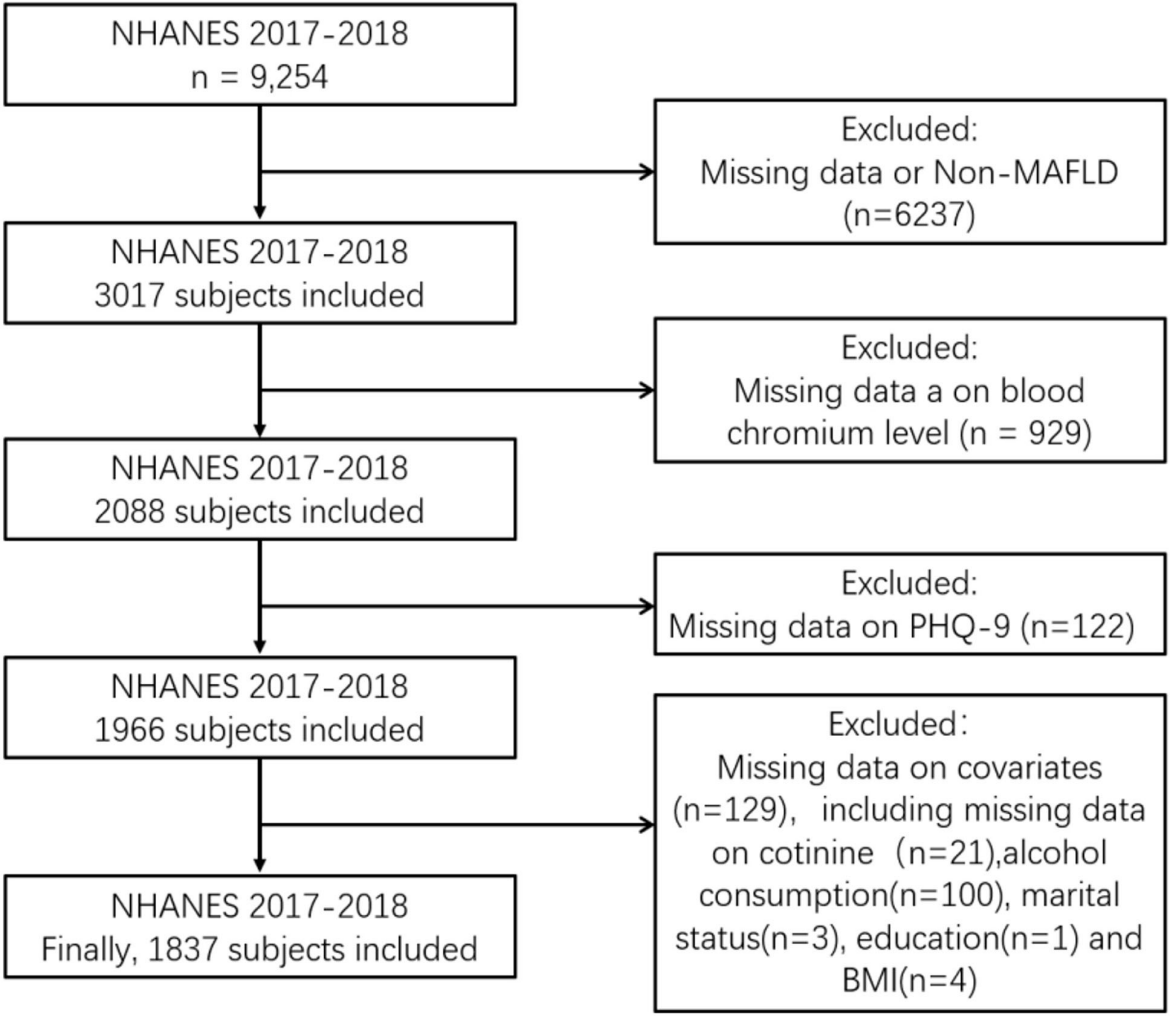
Sample selection flowchart from National Health and Nutrition Examination Survey (NHANES2017-2018).

NHANES is a collection of surveys aimed at evaluating the health and nutritional well-being of the general population living outside of institutions across the entire United States, including the District of Columbia. The National Center for Health Statistics Research Ethics Review Board granted permission for the NHANES study protocol, and all participants were fully informed and gave their consent. The NHANES database provides free and anonymous access to all collected data via https://www.cdc.gov/nchs/nhanes/about_nhanes.htm, which is highly relevant to this study. This study utilized NHANES data from 2017-2018, which included 9,254 subjects in the survey period. The exclusion criteria were non-MAFLD (n=6,237), incomplete serum chromium data (n=929), missing depression data (n=122), no information on marriage (n=3), no information on educational attainment (n=1), no data on cotinine (n=21), no data on alcohol consumption (n=100), and missing body mass index (n=4). The final study population comprised 1,837 respondents (see **Figure 1**).

### 2.2 Definition and assessment

#### 2.2.1 Definition of MAFLD

MAFLD diagnosis involves obtaining evidence from liver biopsy, imaging, and blood biomarker analyses. Hepatic fat accumulation may be suggested by the evidence in conjunction with any one of the following conditions: being overweight/obese, having type 2 diabetes, or experiencing metabolic dysfunction. Metabolic dysfunction is defined as having two or more risk factors for metabolic abnormalities, as follows: 1) For Caucasian individuals, having a waist measurement of at least 102/88 cm in men and women, respectively, or 90/80 cm in Asian men and women; 2) Individuals with a systolic blood pressure of 130 mmHg or higher and/or a diastolic blood pressure of 85 mmHg or higher, or those taking medication to manage hypertension;3) Elevated levels of triglycerides in the bloodstream at or above 150 mg/dL (or 1.70 mmol/L) or requiring specific medication; 4) Plasma HDL-cholesterol < 40 mg/dL (< 1.0 mmol/L) in men and < 50 mg/dL (< 1.3 mmol/L) in women or requiring specific medication; 5) Those with prediabetes (i.e., a fasting glucose level ranging from 100 to 125 mg/dL [5.6 to 6.9 mmol/dL], or from 140 to 199 mg/dL [7.8 to 11.0 mmol/dL] or from HbA1c 5.7 to 6.4% [39 to 47 mmol/dL]) at two hours post-load; 6) Assessment of the insulin resistance index ≥ 2.5 in the steady-state model; 7) Blood hypersensitivity C-reactive protein > 2 mg/L (2).

#### 2.2.2 Evaluation of depression

The mobile clinic utilized a computerized personal interview program administered by skilled interviewers to evaluate the depression levels of the subjects by analyzing their scores on the Patient Health Questionnaire (PHQ-9). Participants were asked to complete the PHQ-9 questionnaire two weeks before their evaluation, and they were required to rate each statement on a scale of 0 to 3, indicating how frequently they experienced each symptom. According to prior research, the participants’ level of depression was classified as either absent (PHQ-9 scores < 10) or present (PHQ-9 scores ≥ 10) (31). The range of PHQ-9 scores observed was between zero and twenty-seven.

#### 2.2.3 Independent variable: blood chromium content

The main independent variable was the level of chromium found in the blood. In NHANES 2017-2018, individuals over the age of 40 provided whole blood samples at MEC. These samples were utilized to measure chromium concentration, with the highest recorded level being 165.76 nmol/L. The minimum detectable concentration (MDC) was determined to be 5.58 nmol/L. For analytes that yielded results lower than the limit of detection (LLOD), an approximate fill value was determined by dividing the LLOD by the square root of 2 (LLOD/sqrt [2]).

#### 2.2.4 Covariates

Studies have indicated a significant association between serum chromium levels and depression, with factors including age, gender, race, education level, marital status, smoking, BMI, and alcohol consumption (22, 32–35). The study considered various factors that could affect the association between serum chromium levels and depression, including age, gender, ethnicity, education level, marital status, smoking habits, alcohol intake, and body mass index. The body mass index was determined by dividing the recorded weight (in kilograms) by the height (in meters) squared. These variables were selected based on their potential to influence the observed relationship between chromium levels and depression.

Educational attainment was categorized as either high school or lower, or higher than high school. Serum cotinine levels were used to categorize smoking into three tiers: minimal (<0.015 ng/mL), moderate (0.015-3 ng/mL), and significant (≥3 ng/mL) (36–38). Two 24-hour dietary recalls were used to gather information on daily alcohol consumption, and alcohol consumption was categorized as either never or having a drinking behavior (37). The measurement of weight in relation to height squared was used to categorize BMI into two groups: those under 30 kg/m² and those at or above 30 kg/m² (39).

### 2.3 Statistical analysis

A weighted chi-square test and weighted linear regression model were used to test the differences between the groups. A logistic regression model was used to assess the relationship between each factor and depression. The association between serum chromium levels and depression in MAFLD patients was analyzed by multivariate binary logistic regression analyses to determine the odds ratio (OR) and 95% confidence interval (CI). Various factors, such as sex, age, ethnicity, education level, marital status, BMI, cotinine, and alcohol consumption, were considered as potential confounders and adjusted for. Furthermore, we conducted subgroup analyses according to the following categories: sex (men, women), age (<65 years old, ≥65 years old), ethnicity (Other Hispanic/Non-Hispanic White, Others), education level (High school or less, College or above), marital status (Married/Living with partner, Divorced/Never married/Separated/Widowed), cotinine (Low, Moderate, High), alcohol consumption (Never, Drinking), and BMI (<30, ≥30). Statistical analysis was conducted using Stata 16 and R software (version 4.2.1, which includes “forest plots”). In all bi-directional statistical analyses, any outcome that had a p-value lower than 0.05 was considered statistically significant.

## 3 Result

### 3.1 General characteristics

We analyzed data from 1837 MAFLD patients (969 males, 868 females) from the 2017-2018 NHANES to investigate the relationship between depression and chromium levels. Of these patients, 165 (8.98%) had a PHQ-9 score ≥10, indicating depression. The first table displays the demographic features of the patient population and distinguishes between groups based on PHQ scores (**Table 1**). Female patients, those who were widowed/divorced/separated/never married, had a higher education level, higher serum cotinine levels, never drank alcohol, and higher BMI had a higher prevalence of depression (all P < 0.05). There was no statistically significant difference in the levels of serum chromium between the two groups (p=0.122).

**Table 1:** General Characteristics of included participants (weight).

	Overall (n=1954)	Without (n=1441)	depression (n=513)	P value
Age(%(95%CI))				0.167
<65 years old	69.85 (66.59-72.93)	69.41 (65.96-72.65)	55.98 (63.87-83.21)	
≥65years old	30.15 (27.07-33.41)	30.59 (27.35-34.04)	25.25 (16.79-36.13)	
Sex(%(95%CI))				<0.001
Male	53.46 (49.82-57.06)	54.92 (51.10-58.69)	37.46 (27.06-49.17)	
Female	46.54 (42.94-50.18)	45.08 (41.31-48.90)	62.54 (50.83-72.94)	
Ethnicity(%(95%CI))				0.076
Other Hispanic/Non-Hispanic White	73.13 (70.41-75.68)	73.68 (70.88-76.30)	67.07 (56.28-76.32)	
Others	26.87 (24.32-29.59)	26.32 (23.70-29.12)	32.93 (23.68-43.72)	
Marital status(%(95%CI))				<0.001
Married/Living with partner	70.95 (67.82-73.91)	72.90 (69.68-75.91)	49.67 (38.37-60.99)	
Divorced/Never married/Separated/Widowed	29.05 (26.10-32.18)	27.10 (24.09-30.32)	50.33 (39.01-61.63)	
Education level(%(95%CI))				0.031
High school or less	59.64 (56.07-63.11)	60.38 (56.63-64.02)	51.50 (40.15-62.70)	
College or above	40.36 (36.89-43.93)	39.62 (35.98-43.37)	48.50 (37.30-59.85)	
Alcohol consumption				<0.001
Never	73.31 (69.85-76.51)	71.99 (68.30-75.41)	87.85 (79.23-92.97)	
Drinking	26.69 (23.49-30.15)	28.01 (24.58-31.70)	12.35 (7.03-20.77)	
Cotinine				<0.001
Low	43.88 (40.23-47.59)	44.69 (40.85-48.60)	35.00 (24.58-47.07)	
Moderate	35.16 (31.87-38.59)	36.19 (32.69-39.83)	23.93 (16.53-33.33)	
High	20.97 (18.20-24.04)	19.12 (16.33-22.27)	41.07 (30.3-52.77)	
BMI(%(95%CI))				<0.001
<30	41.63 (38.07-45.27)	43.17 (39.4-47.02)	24.85 (16.78-35.16)	
≥30	58.37 (54.73-61.93)	56.83 (52.98-60.6)	75.15 (64.84-83.22)	
Serum Chromium Level (M ± SD)	6.51 ± 5.31	6.70 ± 7.23	5.80 ± 1.32	0.122

Mean +/- SD for serum chromium level. The weighted linear regression model was utilized to compute the P value. The weighted chi-square test was utilized to determine the P value.

### 3.2 Impact factors of depression

**Table 2** presents the results of the univariate analysis, which show a significant association between serum chromium levels and depression (OR=0.82, 95%CI: 0.69-0.96, p=0.016). Additionally, gender, marital status, serum cotinine level, alcohol consumption, and BMI were found to be related to depression. Female MAFLD patients had 2.03 times higher odds of depression than male patients (OR=2.03, 95%CI: 1.23-3.36, p<0.001). Individuals in committed relationships, whether married or cohabiting, displayed a reduced likelihood of experiencing depressive symptoms compared to those who were single, divorced, widowed, or separated (OR=2.73, 95%CI: 1.68-4.44, p<0.001). Furthermore, those with high levels of serum cotinine had higher odds of depression (OR=2.74, 95%CI: 1.51-5.00, p<0.001), whereas those who drank alcohol had lower odds (OR=0.36, 95%CI: 0.19-0.69, p=0.002). According to the data, participants with a BMI exceeding 30 are significantly more likely to experience depression than their counterparts with a BMI below 30 (OR=2.30, 95%CI: 1.37-3.86, p=0.002).

**Table 2:** Univariate analysis results for factors associated with depression in MAFLD patients.

	Statistics	PHQ9, β (95%CI)	p
Age			
<60 years old	1206 (65.65%)	Ref.	
≥60 years old	631 (34.35%)	0.77 (0.45-1.31)	0.333
Sex			
Male	969 (52.75%)	Ref.	
Female	868 (47.25%)	2.03(1.23-3.36)	0.006
Race			
Mexican American/Non-Hispanic White	880 (47.90%)	Ref.	
Other Race	957 (52.10%)	1.37(0.85-2.22)	0.194
Marital status			
Married/living with partner	1182 64.34%)	Ref.	
Widowed/divorced/separate/never married	655 (35.66%)	2.73(1.68-4.44)	<0.001
Education level			
High school or less	1024 (55.74%)	Ref.	
College or above	813 (44.26%)	1.44 (0.88-2.33)	0.144
Cotinine			
Low	710 (38.65%)	Ref.	
Moderate	747 (40.66%)	0.84(0.47-1.52)	0.572
High	380 (20.69%)	2.74(1.51-5.00)	0.001
Drinking Behavior		Ref.	
Never	1347 (73.31%)		
Drinking alcohol	490 (26.69%)	0.36(0.19-0.69)	0.002
BMI			
<30	791 (43.06%)	Ref.	
≥30	1046 (56.94%)	2.30(1.37-3.86)	0.002
Serum chromium level (nmol/L)	6.51 ± 5.31	0.82(0.69-0.96)	0.016

β (95%CI) P value/OR (95%CI) P value.

### 3.3 Association between blood chromium level and depression in MAFLD

There was no significant difference in the levels of serum chromium between the two groups (p=0.122) (**Table 3**). The first model, using unadjusted data, demonstrated an inverse association between serum chromium levels and the likelihood of depression (OR=0.82, 95% CI: 0.69–0.96, p=0.016). In the second model, the data were adjusted for several demographic factors, including age, sex, race, education level, and marital status. We obtained similar results (OR=0.83, 95% CI: 0.71–0.96, p=0.016). Finally, in the third model, additional adjustments were made for cotinine, alcohol consumption, and BMI. The inverse association between blood chromium levels and depression persisted within this model (OR=0.83, 95% CI: 0.71–0.97, p=0.021).

**Table 3:** Relationship between blood chromium and depression in different models.

	OR	95%CI	p
Model1	0.82	0.69-0.96	0.016
Model2	0.83	0.71-0.96	0.016
Model3	0.83	0.71-0.97	0.021

Data in model 1 were unadjusted; Model 2 were adjusted for age, sex, race, education level, marital status; Model 3 was further adjusted for age, sex, race, education level, marital status, cotinine, alcohol consumption, BMI.

### 3.4 Subgroup analyses

**Figure 2:**
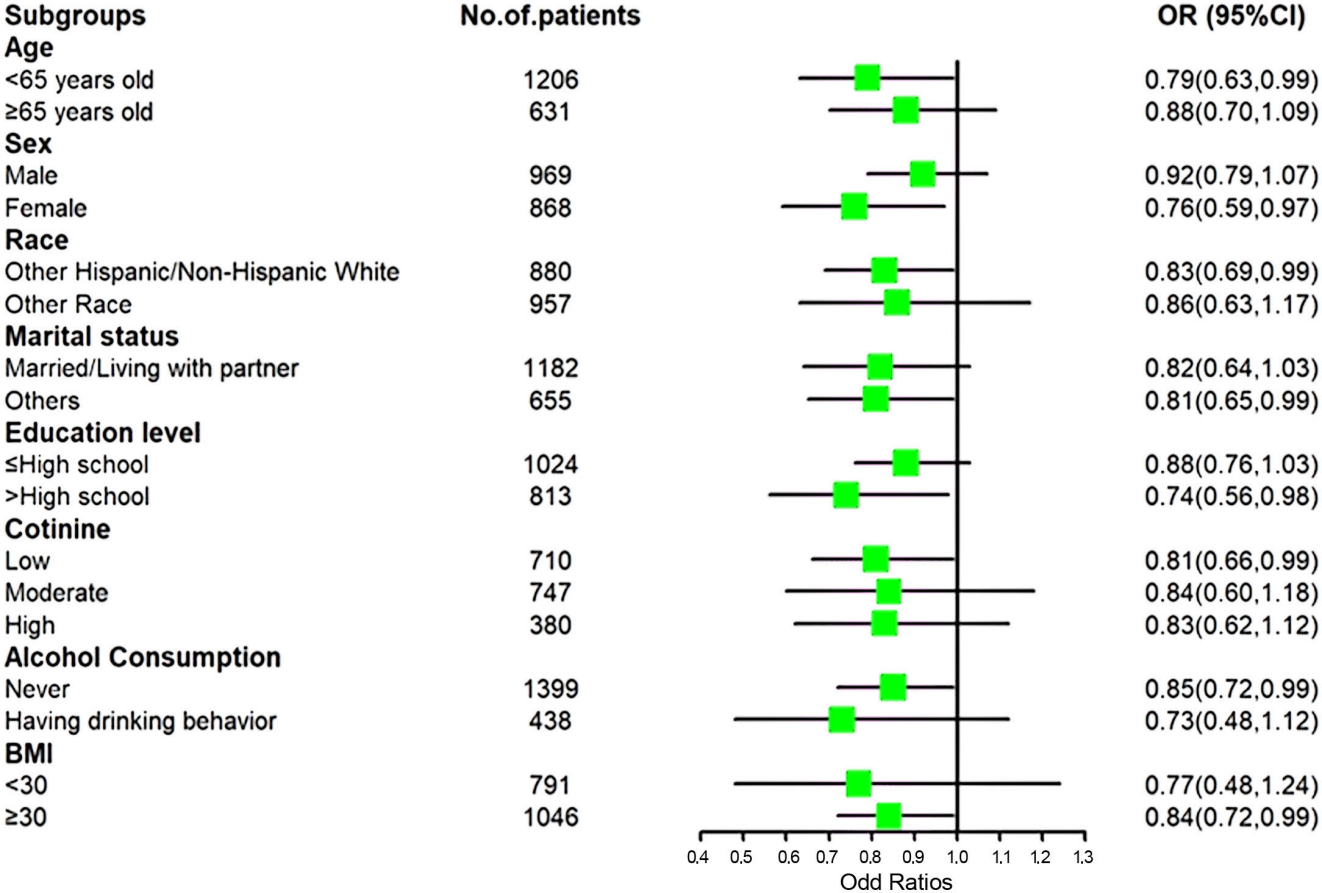
The association between serum chromium and depression by stratisfied analyses.

Among patients who were women, married, college-educated or above, and classified as obese (BMI ≥ 30), lower levels of depression were associated with higher levels of blood chromium, even after adjusting for age, sex, ethnicity, marital status, education level, serum cotinine, and alcohol consumption (**Figure 2**).

## 4 Discussion

After accounting for possible influencing factors, our examination of NHANES 2017-2018 data indicated an inverse association between the risk of depression in patients with MAFLD and serum chromium levels. This finding is consistent with previous research that has shown an association between chromium and depressive symptoms. Additionally, our analysis supports the relationship between serum chromium and depression.

Fatty acid sources from white adipose tissue, *de novo* lipogenesis, and remnants of triglyceride-rich lipoproteins contribute to excessive hepatic triglyceride synthesis in MAFLD (40), suggesting that insulin resistance may be a key driver of the disease. Interestingly, insulin resistance also plays an important role in the development of major depressive disorder (41). A prospective study with elderly men found that men with insulin resistance were more likely to develop depressive symptoms, with a risk ratio of 2.3 over a 5-year follow-up period (42). Another Dutch cohort study showed that insulin resistance positively predicted incident major depressive disorder over a 9-year follow-up period among adults with no history of depression or anxiety disorder (43).

Chromium has been recognized for enhancing insulin sensitivity in the hypothalamus, promoting the function of serotonin, melatonin, and dopamine, thereby effectively treating neurobehavioral processes like depression and comorbidity (44). Insulin resistance is closely linked to hepatic steatosis (25) and has been implicated in the mechanisms of depression in individuals with depression (26). Under conditions of high blood sugar, chromium has been shown to decrease the expression of proteins that contribute to insulin resistance, as well as prevent the release of pro-inflammatory cytokines and the process of lipid peroxidation (45, 46). Both humans and animals have exhibited reduced endocrine responses to 5-HT2A receptor stimulation following the administration of chromium over a short period of time (47, 48). Numerous clinical studies have demonstrated that chromium possesses antidepressant properties (26, 32, 44). Our research also discovered that individuals diagnosed with MAFLD showed a tendency of increased levels of serum chromium with reduced depression symptoms, suggesting a possible link between higher blood chromium levels and a lower incidence of depression. This evidence supports chromium’s potential as a therapeutic option for comorbid depression in MAFLD patients.

Subgroup analysis revealed an inverse association between serum chromium and depression in certain populations with MAFLD, including women, white individuals, those with lower levels of education, non-drinkers, and individuals with heavier body mass. The result may be because of the higher prevalence of depression in these populations. A longitudinal Dutch study with 1269 adult participants found that among participants with current MDD, IR was positively associated with depression severity, while among participants with remitted MDD, neither depression severity nor chronicity was associated with IR (49). As chromium acts as an insulin sensitizer, it can reverse insulin resistance (50). Therefore, the results in our study showed an association between higher serum chromium levels and a lower risk of depression in individuals at high risk of depression, while the subgroups with a relatively lower prevalence of depression did not exhibit this association. Additionally, a cross-sectional survey using data from the 2015-2016 US NHANES survey found no association between serum chromium and depression in either men or women (44). This may be due to the characteristics of the sample - healthy individuals have minimal insulin resistance symptoms. In the absence of significant insulin resistance, we cannot find an association between depression and chromium. A clinical study with 95 adults experiencing a major depressive disorder episode showed that IR reduced in treatment responders, conversely exacerbation of IR during antidepressant treatment mediated non-response (51). We infer that the effective treatment of depression with chromium may require additional conditions, such as being limited to individuals with insulin resistance. This may also explain why chromium supplementation is an efficient therapy for atypical depression. Further research is needed to detect the relationship between chromium, depression, and insulin resistance to provide supporting evidence.

Our study indicates that the prevalence of depression among individuals with MAFLD in the US is 8.98%, which is considerably higher than the depression rate in the general population (52). This finding is in line with recent research that highlights a notably higher incidence of depression in individuals with MAFLD compared to those without the condition (15). Given that MAFLD may act as a chronic stressor, it may lead to a negative feedback loop between MAFLD and depression. These results emphasize the significance of recognizing depression as a comorbidity in individuals with MAFLD, as it may have important implications for their management and treatment.

According to our research, individuals with MAFLD who were not in a marital or cohabiting relationship were found to have a significantly higher prevalence of depression, with a nearly 150% higher occurrence compared to those who were in a committed partnership. This suggests that marriage or having a partner may be a protective factor against depression in individuals with MAFLD, as supported by previous research (53). Additionally, unmarried, and non-cohabiting adults have previously reported higher rates of depression (54). Although prior research has indicated that individuals with higher education levels experience less depression, our examination of individuals with MAFLD did not reveal any notable variations in depression rates depending on their education level. It is possible that the impact of obesity, MAFLD symptoms, and other factors on depression outweighed the influence of education in this population. In contrast, we found a higher prevalence of depression in individuals with a BMI over 30 and those with higher serum cotinine levels. Our examination indicated that people who engage in drinking have a lower likelihood of experiencing depression compared to those who were non-drinkers. This finding is in line with previous studies. A recent study conducted a secondary analysis of the National Longitudinal Survey of Youth 1979 cohort and employed a marginal structural model (MSM) approach to investigate the association between alcohol consumption and depression. It found that both consistent occasional and consistent moderate drinkers were predicted to have reduced depressive scores and lower possible depression at age 50 compared to non-drinkers (55). Another study examined the association between the Mediterranean diet and depressive symptoms and found that moderate drinkers, compared to non-drinkers, were associated with lower depression and anxiety severity and lower odds of being currently depressed (56). This may be because drinking alcohol in moderation has been linked to a decrease in health hazards, such as the likelihood of experiencing depression (15, 37). For individuals with MAFLD, moderate alcohol consumption may have positive effects, including stress reduction and improved socialization.

However, there are certain constraints to our research that should be acknowledged. To begin with, this study is of an observational nature and therefore unable to determine a cause-and-effect relationship. Future research using a longitudinal study design is necessary to confirm the causal relationship between blood chromium and depression. Secondly, unmeasured confounders may have influenced the observed associations. Thirdly, not assessing prior antidepressant use is a limitation, as it is possible that this could have impacted the occurrence of depression. Fourthly, using the self-reported PHQ-9 questionnaire for depression assessment may have led to inaccurate reporting of symptoms. Moreover, the PHQ-9 score alone cannot be used to diagnose depression but can screen for depressive symptoms. Therefore, in this study, we can only explore the relationship between depressive symptoms and blood chromium levels. Lastly, patients with MAFLD had serum chromium levels below the minimum concentration required for examination, resulting in the use of an imputed fill value in the analyte results field.

## 5 Conclusion

This research marks one of the first endeavors to investigate the association between depression and blood chromium levels in individuals with MAFLD. Individuals diagnosed with MAFLD have a higher likelihood of experiencing depression, whereas increased levels of chromium in the bloodstream are associated with a decreased risk of developing depressive symptoms. Moreover, the negative relationship remained consistent even after taking into consideration any possible factors that could have affected the results. Recognizing the connection between serum chromium and depression could have practical implications for treating patients with MAFLD, such as supplementing with chromium as a potential therapy for depression.

## Data availability statement

The original contributions presented in the study are included in the article/supplementary material. Further inquiries can be directed to the corresponding author.

## Ethics statement

The studies involving humans were approved by The National Center for Health Statistics Research Ethics Review Board. The studies were conducted in accordance with the local legislation and institutional requirements. The participants provided their written informed consent to participate in this study.

## Author contributions

XL: Conceptualization, Supervision, Writing – original draft. XX: Formal analysis, Investigation, Writing – original draft. BJ: Formal analysis, Writing – review and editing. YY: Supervision, Writing – original draft, Data curation, Resources. FD: Conceptualization, Writing – original draft, Supervision, Methodology, Visualization. SQ: Conceptualization, Investigation, Writing – original draft, Writing – review & editing.
